# Supplementary material for: Comparative genomics provides new insights into the diversity, physiology, and sexuality of the only industrially exploited tremellomycete: Phaffia rhodozyma
Source: BMC Genomics. 2016 Nov 9;17:901. doi: 10.1186/s12864-016-3244-7 (PMC5103461; doi:10.1186/s12864-016-3244-7)
Supplement: Additional file 6: — List of orphan genes with links to PFAM (related to Additional file 1: Table S1). (ZIP 1428 kb) [file 12864_2016_3244_MOESM6_ESM.zip › PFAM_UNIQUE/G00304_P.html]

#                                                                            --- full sequence --- -------------- this domain -------------   hmm coord   ali coord   env coord
  
# target name        accession   tlen query name           accession   qlen   E-value  score  bias   #  of  c-Evalue  i-Evalue  score  bias  from    to  from    to  from    to  acc description of target
  
#------------------- ---------- ----- -------------------- ---------- ----- --------- ------ ----- --- --- --------- --------- ------ ----- ----- ----- ----- ----- ----- ----- ---- ---------------------
  
G00304\_P             -           1051 Herpes\_BLLF1         PF05109.8    830       0.3    7.6   5.2   1   1    0.0016      0.72    6.4   5.2   574   664   957  1047   927  1049 0.83 - Herpes virus major outer envelope glycoprotein (BLLF1)  
G00304\_P             -           1051 PTRF\_SDPR            PF15237.1    246       2.2    6.3   5.3   1   1    0.0067       4.5    5.3   5.3   160   219   832   891   810   907 0.75 - PTRF/SDPR family
